# Management of periprosthetic humeral fractures after shoulder arthroplasty: A case report on open reduction and internal fixation with strut allograft

**DOI:** 10.1016/j.tcr.2026.101350

**Published:** 2026-04-27

**Authors:** Alexis Kapitanov, Alain Akiki, Geoffroi Lallemand

**Affiliations:** Department of Orthopedic Surgery, Hospital Riviera Chablais, Route du Vieux Séquoia 20, 1847, Rennaz, Switzerland

**Keywords:** Periprosthetic humeral fracture, Shoulder arthroplasty, Open reduction and internal fixation, Strut allograft, Case report

## Abstract

**Introduction:**

This case report details the management of a periprosthetic humeral shaft fracture treated by open reduction and internal fixation (ORIF) with a strut allograft. To date, only 50 patients treated with strut allografts for periprosthetic humeral fractures are documented in the literature. Four case series, two retrospective reviews, and one prospective review have been analyzed. Of these, four studies (40 patients) used strut allografts with plates, and three studies (10 patients) used strut allografts with cerclage alone. Fracture union occurred between 3 and 7 months postoperatively. This is the first case report of such a fracture treated with a strut allograft and documented follow-up nearing two years.

**Case report:**

A 77-year-old man with multiple prior right shoulder surgeries, including reverse shoulder arthroplasty in 2017, sustained a proximal periprosthetic humeral fracture in 2023 after a fall. Initial ORIF with a long plate failed due to early loss of reduction. A revision procedure was performed using a longitudinally split fibular strut allograft, with halves placed medially and anteriorly, secured by fiber wires and plate fixation. At two years post-op, the patient was pain-free and functional, with radiographs showing fracture consolidation and graft integration.

**Conclusion:**

Seven studies have reported outcomes of strut allografts in periprosthetic humeral fractures. Only one compared ORIF with and without allograft, finding no outcome difference. Strut allografts are a valuable option for failed ORIF, providing both biological and mechanical stability.

**Level of evidence:**

Level IV.

## Highlights

• First case report of periprosthetic humeral fracture treated with strut allograft and followed for nearly two years.
• Successful revision surgery with fibular allograft after failed ORIF, achieving fracture union and graft incorporation.
• Excellent functional outcome with a Constant Shoulder Score of 84, surpassing previously reported averages.
• Strut allograft provided both mechanical and biological stability in a complex revision shoulder arthroplasty case.

## Introduction

The increased rate of shoulder arthroplasty and rise in life expectancy increased the rate of periprosthetic humeral fractures [1], [2], [3], [4]. In the systematic review Mourkus et al. reported a 75% increase in the number of revision shoulder arthroplasties being undertaken between 2013 and 2019, while revision shoulder arthroplasties for periprosthetic fracture increased by 133% based on National Joint Registry [4]. The incidence of periprosthetic humeral fractures varies between 0,5% and 3% in all shoulder arthroplasty [2], [3], [5], [6], [7], [8], [9]. It represents 11% of all complications related to total shoulder arthroplasty [5], [8]. The risk factors for periprosthetic fracture include weak bones, advancing age, female sex, diabetes and rheumatoid arthritis [3], [5], [9].

We describe a case of a periprosthetic humeral shaft fracture treated with a strut allograft at our hospital. To our knowledge, only 50 patients treated for periprosthetic humeral fractures with strut allografts have been documented in the literature [5], [10], [11], [12], [13], [14], [15]. Among these, only four studies, encompassing a total of 40 patients, used strut allografts with plates, as in our study. The longest follow-up reported in the literature was 14 months [12]. This case report is unique as it is the only one that describes patient outcomes with nearly two years of follow-up.

## Case presentation

The patient is a 77-year-old right-hand dominant man with a past medical history of Bankart open surgical stabilization in the 1980s and rotator cuff surgery in 2000 on his right shoulder. He underwent reverse total shoulder arthroplasty in 2016, which was complicated by an infection treated with a two-stage revision involving prosthesis removal and spacer implantation in 2017. His last shoulder operation was in 2017, with spacer removal and implantation of a reverse shoulder prosthesis (Lima SMR Shoulder with metal back small axioma, 40 mm glenosphere, SMR rod 22, reverse liner medium 40 mm, extension humeral body).

**Fig. 1:**
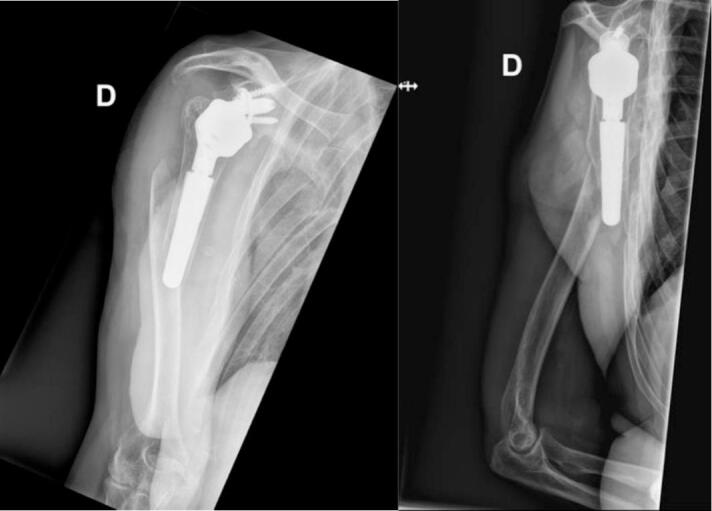
Initial X-rays showing the periprosthetic humeral shaft fracture.

On April 3, 2023, the patient fell from standing height onto his upper right limb, resulting in a proximal periprosthetic fracture of his right humerus. He was initially treated at a small community hospital, where the fracture was classified as a CH2S Kirchoff fracture and a B1 Unified Classification System fracture. His upper limb was immobilized with a humeral sling until the operation. Initial X-rays after the fall are shown in Fig. 1.

Clinically, the patient was referred to our hospital 2 weeks after the fall. He presented with a large hematoma on the lateral side of his humerus without skin breakage. He had no new neurovascular injury following the fall. Prior to the fall, he had hypoesthesia in the fourth and fifth fingers' volar parts, explained by cubital tunnel syndrome.

**Fig. 2:**
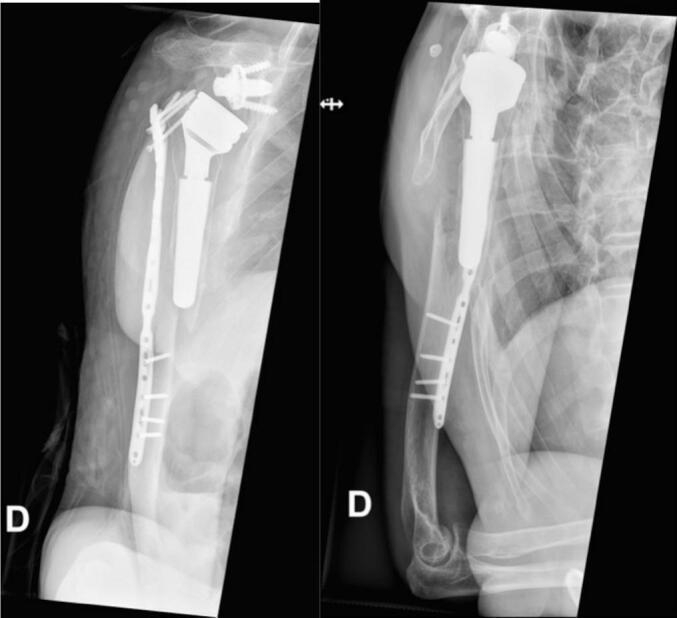
Postoperative radiographs showing the early loss of reduction after ORIF of the humeral periprosthetic fracture.

The patient was treated with ORIF of the right humerus using a long ALPS Zimmer Biomet plate on April 17, 2023 (two weeks after the fall). The operation was performed under general anesthesia and an interscalene block. The patient was positioned in a beach chair position during the intervention. A deltopectoral and anterior humeral approach was used, revealing a spiral fracture. The prosthetic stem was stable in the proximal fragment. Open reduction was performed and held in place with a clamp. The plate was applied with five proximal screws in the major tubercle, one screw in the oblique hole, and three locking distal screws. Final intraoperative X-rays showed satisfactory reduction. Postoperatively, the patient was immobilized with a humeral sling. Postoperative X-rays showed early loss of reduction (Fig. 2). The patient was discharged on April 19, 2023, and revision surgery was planned for April 27, 2023.

**Fig. 3:**
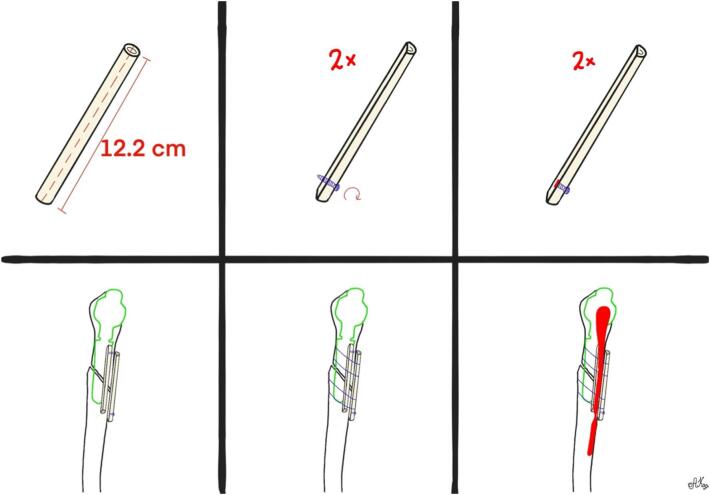
Part 1: A 12.2 cm fibular strut allograft cut longitudinally. Part 2: A screw placed in each part of the fibular strut. Part 3: The distal part of the screw cut flush with the bone. Part 4: One fibular allograft positioned anterior to the fracture line and the second one positioned medial to the fracture site. Part 5: The allografts held in place with four fiber wires passed around the humerus. Part 6: The plate refixed in the proximal fragment.

**Fig. 4:**
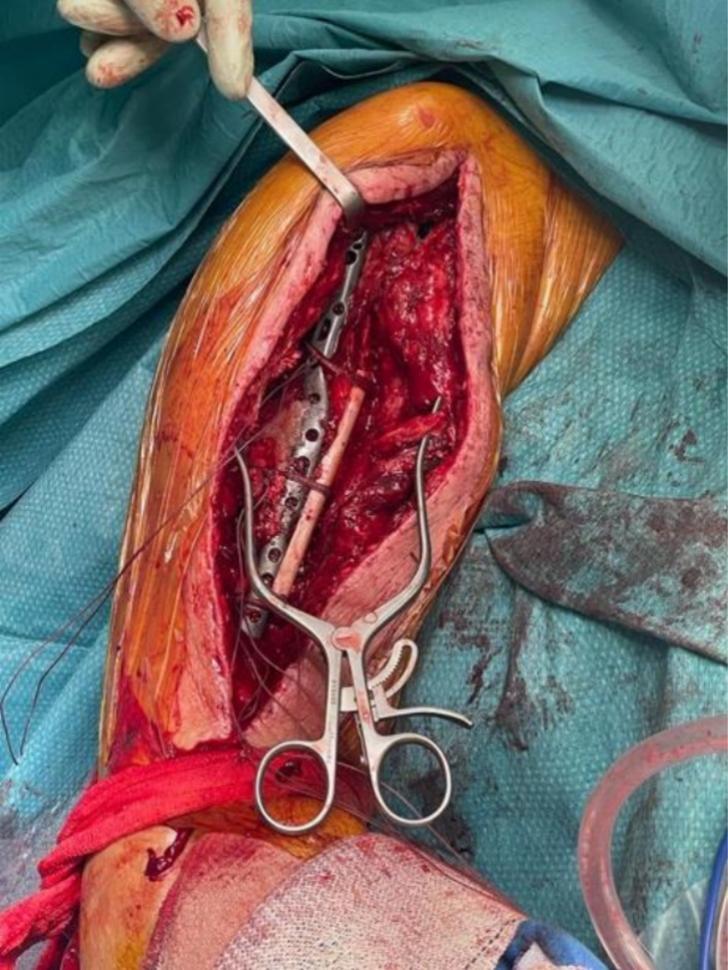
Intraoperative photos taken during revision surgery.

**Fig. 5:**
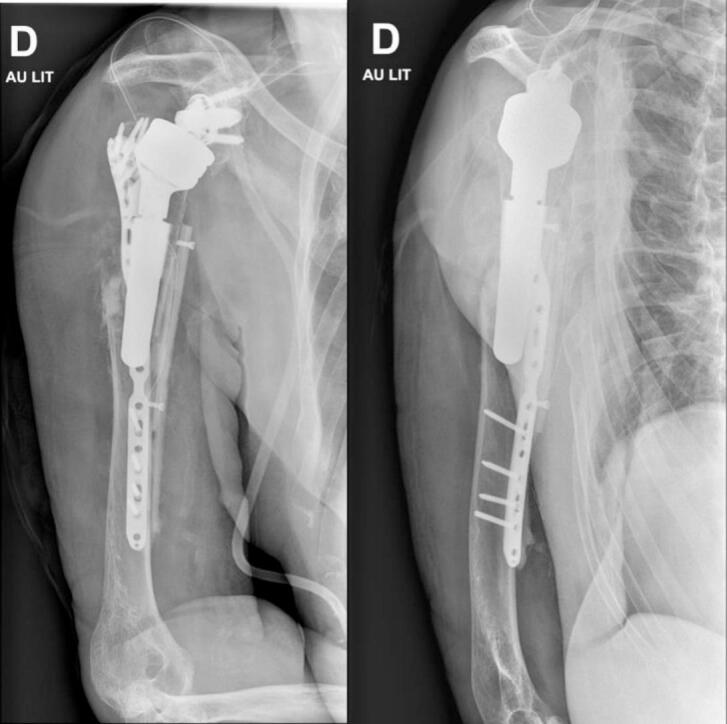
Postoperative X-rays after revision ORIF for periprosthetic humeral fracture.

The revision surgery was performed on April 27, 2023 (10 days after the initial operation). The operation was done under general anesthesia and an interscalene block. The patient was positioned in a beach chair position. A deltopectoral and anterior approach was used again. A serum sample was taken during the operation and sent for culture. During the operation, it was observed that three proximal screws had moved laterally. Open reduction was performed and held in place with a clamp. A 12.2 cm fibular strut allograft was cut longitudinally (Fig. 3, part 1). A screw was placed in each part of the fibular strut, and the distal part of the screw was cut flush with the bone (Fig. 3, parts 2 and 3). These screws were used to localize each fibular strut on X-rays. One fibular allograft was positioned anterior to the fracture line, and the second one was positioned medial to the fracture site (Fig. 3, part 4). They were held in place with four fiber wires passed around the humerus flush to the bone to avoid injury to the radial nerve (Fig. 3, part 5). During this maneuver, the radial nerve was palpated by the surgeon. We added 20 cc of Bonalive Putty to the fracture site (a bone graft substitute with osteoconductive and osteostimulative functions [16]). The plate was refixed in the proximal fragment (Fig. 3, part 6). The choice of a fibular allograft length was based on the patient's humeral anatomy. Four FiberWires were chosen to ensure stable fixation of the graft, with no mobility observed during intraoperative mobilization of the shoulder. The graft was positioned to prevent anterior and medial displacement of the fracture, which was not adequately protected by the lateral plate. Intraoperative X-rays showed satisfactory reduction. Intraoperative photos are shown in Fig. 4. Postoperatively, the patient was immobilized with a humeral sling, with free movements of the elbow and wrist allowed, as well as pendular movements of the shoulder. The postoperative X-ray is shown in Fig. 5. Intraoperative samples sent for bacterial culture were negative, and the patient was discharged from our hospital on April 30, 2023.

**Fig. 6:**
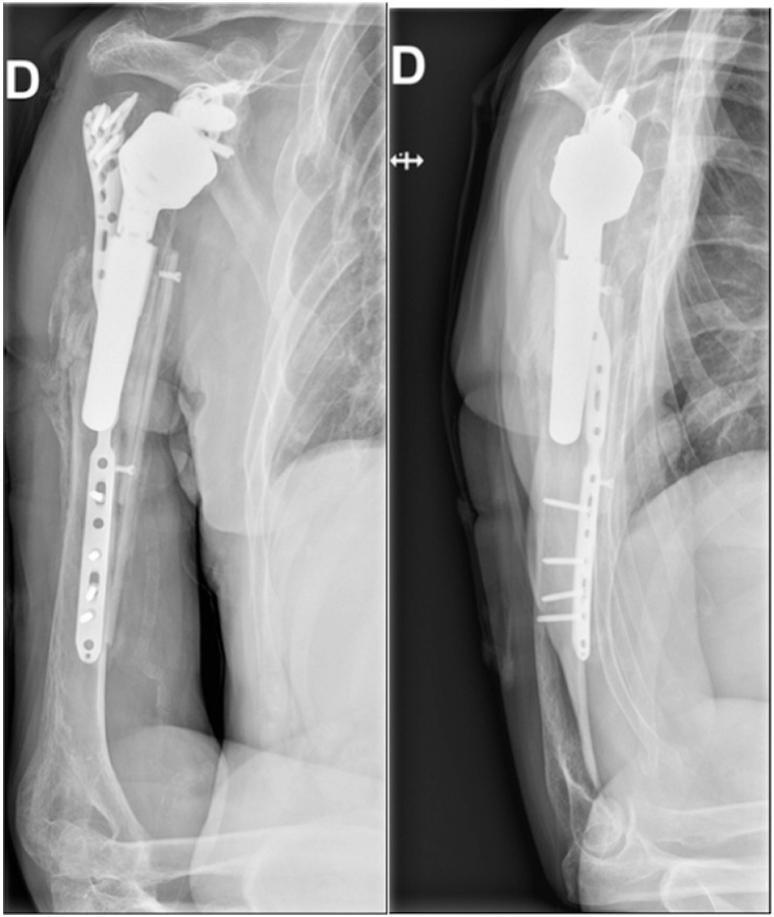
Six weeks postoperative X-rays.

Postoperatively, the patient was seen six weeks after the operation (June 14, 2023). He had no functional complaints and adhered to the postoperative rehabilitation protocol. The visual analog score was 2/10 points. The shoulder subjective score was 20%. The scar presented a small central discharge. Periscapular muscles had normal trophicity. Active and passive elevation were 20° and 20°, respectively (160° in the contralateral shoulder), abduction 20° and 20°, respectively (90° in the contralateral shoulder), external rotation 0° and 0°, respectively (45° in the contralateral shoulder), and internal rotation to L1 (L1 in the contralateral shoulder). The deltoid muscle was competent with no sensory loss. X-rays are shown in Fig. 6, indicating no secondary displacement and the beginning of consolidation. The shoulder sling was discontinued during this postoperative visit, and the patient started physiotherapy with active assisted motion of his shoulder.

**Fig. 7:**
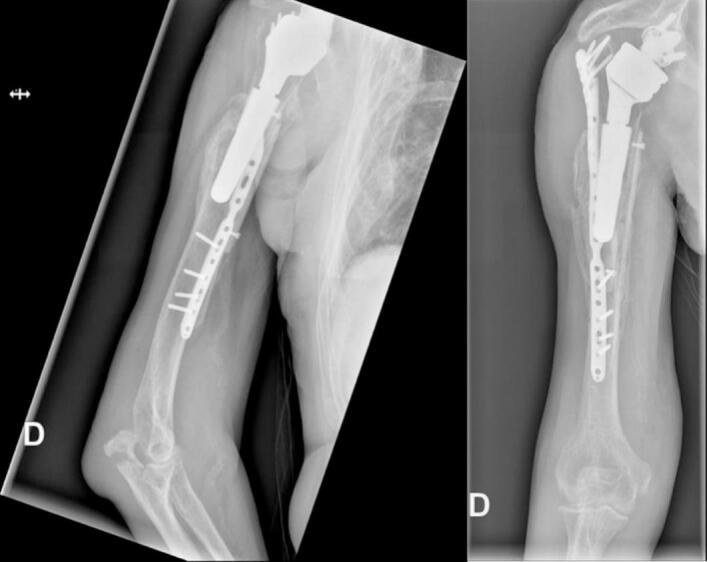
Six months postoperative X-rays after revision ORIF for periprosthetic humeral fracture.

The patient was seen again six months after the operation, on October 25, 2023. He had no functional complaints and adhered to the postoperative rehabilitation protocol. On October 23, 2023, he sustained a direct trauma to his elbow, resulting in a new olecranon fracture. He refused any surgical treatment and immobilization for the olecranon fracture. We advised him to avoid flexion of more than 90° and extension against resistance for the next six weeks. His visual analog score was 0/10 points, the shoulder subjective score was 60%, and the Constant Shoulder Score was 70 points. The scar was dry, and the periscapular muscles had normal trophicity. The deltoid muscle was competent with no sensory loss. X-rays are shown in Fig. 7. The patient continued physiotherapy.

**Fig. 8:**
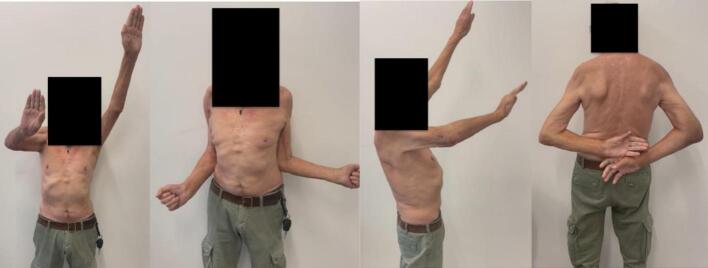
Nine months postoperative clinical status after periprosthetic humeral fracture, showing abduction, external rotation, elevation, and internal rotation.

**Fig. 9:**
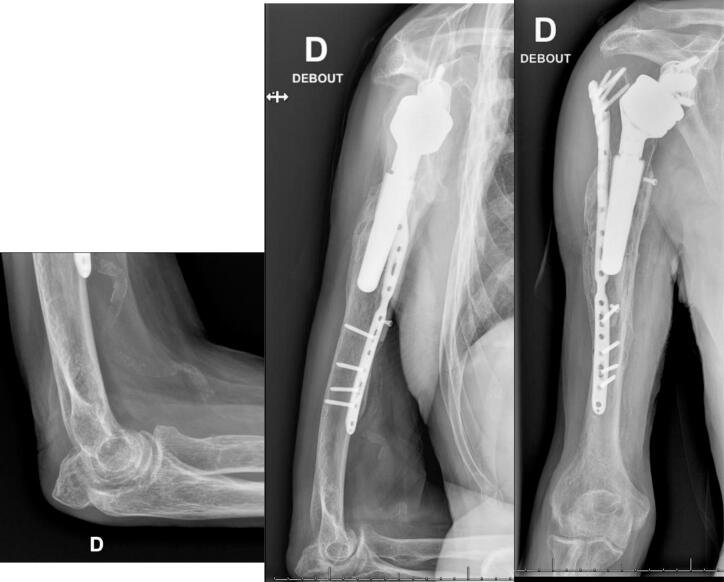
X-rays two years after the operation showing fracture consolidation with graft incorporation. Olecranon fracture showing radiological signs of union.

The last follow-up of this patient was conducted almost two years after the operation. The patient had no functional complaints. The visual analog score was 0/10 points. The shoulder subjective score was 70%, and the Constant Shoulder Score was 84 points (*76 is considered normal for a man of this age*). The scar showed no inflammation and was not adherent. Periscapular muscles had normal trophicity. Active and passive elevation were 90° and 110°, respectively (160° in the contralateral shoulder), abduction 75° and 75°, respectively (90° in the contralateral shoulder), external rotation 20° and 20°, respectively (45° in the contralateral shoulder), and internal rotation to L1 (L1 in the contralateral shoulder) (Fig. 8). X-rays at the two-year follow-up are shown in Fig. 9, indicating fracture consolidation with graft incorporation. The olecranon fracture showed radiological signs of union, and no functional limitation were observed. The evoluation of the patient's mobility and scores can be seen in Table 1. No additional imaging was performed at this point, as the patient had an excellent Constant Shoulder Score and X-rays showed fracture consolidation with graft incorporation.

**Table 1:** Evolution of the patient's mobility and clinical scores.

Follow-up time	6 weeks	6 months	2 years	Contralateral shoulder
Visual analog score	2	0	0	–
Shoulder subjective score (%)	20	60	70	–
Constant shoulder score	57	70	84	–
Active elevation (°)	20	90	90	160
Passive elevation (°)	20	110	110	160
Active abduction (°)	20	75	75	90
Passive abduction (°)	20	75	75	90
Active external rotation (°)	0	20	20	45
Passive external rotation (°)	0	20	20	45
Internal rotation	L1	L1	L1	L1

## Discussion

As mentioned before, only seven studies have described the treatment of periprosthetic humeral fractures using strut allograft, totaling 50 cases [5], [10], [11], [12], [13], [14], [15]. These include four case series, two retrospective reviews, and one prospective review [5], [10], [11], [12], [13], [14], [15].

Various scores were used in these studies. The Constant Shoulder Score (CSS) was used by Rollo et al. (mean score 66.7) [13], Thés et al. (25.1) [14], and Martinez et al. (64) [12]. Rollo et al.'s study, the largest with 15 patients, reported a CSS lower than ours (84 in our study), possibly due to our longer follow-up of nearly two years. The average follow-up was 12 months, with two studies not specifying the duration [5], [10], [11], [12], [13], [14], [15].

Other scores included the American Shoulder and Elbow Surgeons score (ASES) used by Giovanni et al. (mean score 73) [15] and Thés et al. (46.5) [14]. Greiner et al. did not use clinical scores [10], while Kumar et al. [11]. used the Neer and Confield scores with unsatisfactory results. Mourkus et al.'s systematic review reported an average ASES score of 55.53 and CSS of 44.75 [4]. Due to the different scores used, direct comparison is challenging.

Mourkus et al. highlighted a lack of uniformity in classifying periprosthetic fractures and reporting outcomes [4]. The Wright and Cofield classification was most used (15 studies), followed by Worland et al. (5 studies), O'Driscoll et al. (5 studies), Campbell et al. (4 studies), the Unified Classification System (UCS) of Duncan and Haddad (2 studies), and Groh et al. (1 study) [4]. Twelve studies used no classification system. In our review, five publications used Worland et al.'s classification and two used Wright and Cofield's. Fractures treated with strut allograft were mostly at or distal to the stem, with two involving unstable stems.

Mourkus et al. suggested using the UCS for better reporting [4]. We believe the Kirchkoff classification is the most comprehensive for periprosthetic humeral fractures, though the UCS is simpler and could facilitate registry reporting.

Several surgical techniques for using strut allograft in periprosthetic humeral fractures are described. Four studies (40 patients) used strut allograft with plates, and three studies (10 patients) used cerclage without plate augmentation [5], [10], [11], [12], [13], [14], [15]. Our study used strut allograft with plate augmentation. Comparing outcomes is difficult due to study heterogeneity, and it often depends on surgeon preference. Rollo et al. suggested that revision to reverse shoulder prosthesis with or without strut allograft is safe and appropriate, with no significant difference between groups [13]. In our experience, strut allograft is valuable in failed open reduction and internal fixation, providing both biological and mechanical stability.

Complications included radial nerve neuropraxia (resolved), wound dehiscence (resolved with irrigation and debridement), intra-operative fracture, lateral arm pain, shoulder function limitation, and one death after one year [5], [10], [11], [12], [13], [14], [15]. Strut allograft is often used for more complex fractures, possibly explaining these complications. Our case had no complications post-operation, and the olecranon fracture was unrelated.

Fracture union occurred between 3 and 7 months post-operation. Two studies showed no graft incorporation, one showed incorporation between 6 and 13 months, and four did not mention graft incorporation. Our case showed fracture union at 3 months and graft incorporation at 2 years, likely due to longer follow-up.

This is the first case report of periprosthetic humeral fracture with strut allograft and nearly two years of follow-up. The study's limitation is the literature's heterogeneity, as noted in previous systematic reviews [4]. Few patients were treated with strut allograft, complicating outcome comparisons. Rollo et al.'s study, the only one comparing techniques, found no difference [13]. All reviewed studies suggest strut allograft is a useful option for treating periprosthetic humeral fractures. Table 2 summarizes studies on the use of allograft in periprosthetic humeral fractures.

**Table 2:** Summarizes studies on the use of allograft in periprosthetic humeral fractures.

First author	Year of publication	Country	Number of patients	Type of study	Follow-up (in mounths)	Clinical scores	Type of fracture	Surgical treatment	Complications	Time to union	Time to graft incorporation	Conclusion
Vicenti [15]	2022	Italy	18	Prospective review	12	American Shoulder and Elbow Surgeons score 73	7 B2 and 11C Worland	Posterior humeral approach, posterior cortex plate fixation, anterior strut allograft, screws, and cerclage wires	2 patients with wound dehiscence, resolved with a superficial irrigation and debridement	4,2 months	No information	Posterior approach with a posterior plate placement and anterior strut allograft could be a good treatment option for periprosthetic humeral fractures
Rollo [13]	2020	Italy	30: −15 with stut allograft −15 without stut allograft	Multicentric retrospective cohort	12	Constant Shoulder Score 66.7 in both groups with no statistical difference between the groups, Oxford Shoulder Score (OSS)	For 2nd group: B1 in 4 patients, B2 in 6 patients, B3 in 1 patient, C in 4 patients by Worland	First group 15 patients underwent plate, ring, screws, and strut allografts. Second group of 15 patients was treated with only plates and screws.	Blood loss of 474 ± 229.01 mL, intra operative fracture in one (6.67%), death after one year of follow up in one (6.67%) patient	For 2nd group: 122.7 days = 4 months	No information	Revision to reverse shoulder prosthesis with a long-stem implant with or without cortical strut allograft augmentation to be safe and appropriate
Thés [14]	2017	France	6	Case series	10	Visual analog scale (VAS) 4.2, functional American shoulder and elbow surgeons score (ASES) 46.5 and Constant Shoulder Score (CSS) 25.1	4 patients with type B1 and 2 patients with type C by Worland	Reduction of the fractures, two hemicylinder tibial allografts as long as possible were placed around the humerus. The allograft was fixed with two cerclages wires in a sarcophagus manner.	Radial paralysis occurred in one patient in the immediate postoperative period with full recovery	6 months	No graft resorption during follow-up	Cortical onlay strut allograft can be used for internal fixation of periprosthetic fractures of the humerus without loosening of the stem component in patients with osteoporotic bones and bone loss, with positive results
Trompeter [5]	2012	UK	3	Case series	12	Oxford shoulder score (OSS) and disabilities of the arm, shoulder, and hand score (DASH), returned to pre-injury level, no information on score	Type B2 2 patients by Worland and primary spiral fracture below malunited neck of humerus fracture	Reverse geometry long-stem implant and augmentation with biological cortical strut allograft and cables	Radial nerve neurapraxia that resolved entirely	3–7 months	6, 8 and 13 months	Revision to reverse geometry long-stem implant with or without cortical strut allograft augmentation to be safe and appropriate in the management of these complex injuries, although technically challenging, and has excellent initial to medium-term results
Martinez [12]	2011	Spain	6	Case series	14	Constant Shoulder Score 64,	6 patients with type C by Cofield	Open reduction and internal fixation with plate and strut allograft augmentation	Pain in the lateral area of the arm	5,4 months	No information	Internal fixation with plate, cable wires and strut allograft augmentation achieve satisfactory results for periprosthetic humeral fractures
Greiner [10]	2011	Germany	7, 1 with strut allograft	Case series	No information	No information	Type B3 by Worland	Fracture was stabilized and a long-stemmed prosthesis with a CTA Head was implanted. Due to poor bone stock, additional augmentation with a femoral strut graft and a 3.5 angular stable plate was performed	No pain but important limitation of the shoulder function	No information	No information	The use of additional allo- or autogeneous bone grafting is recommended in the current literature.
Kumar [11]	2004	US	16; 1 with stut allograft	Retrospective review	No information	Systems of Neer et al. and Cofield.	Type A by Cofield	Open reduction internal fixation with use of screws, cables, and a cortical humeral strut allograft	Unsatisfactory clinical result with limited external rotation and abduction, occasional moderate pain	4 months	No graft incorporation	Supplementary fixation with an allograft or with a plate and screws and cables can be used to obtain secure fixation for this type A fracture

## Conclusion

Seven studies have reported the outcomes of using cortical strut allografts for periprosthetic humeral fractures [5], [10], [11], [12], [13], [14], [15]. Only one study compared the use of strut allografts with ORIF to ORIF without strut allografts, finding no significant difference in outcomes [13]. In our experience, the use of strut allografts is a valuable option in cases of failed ORIF, as it provides both biological and mechanical stability to the fracture. This case report highlights the successful use of a fibular strut allograft in achieving fracture consolidation and graft incorporation, demonstrating its potential as an effective treatment strategy for complex periprosthetic humeral fractures.

## CRediT authorship contribution statement

**Alexis Kapitanov:** Conceptualization, Writing – original draft. **Alain Akiki:** Validation. **Geoffroi Lallemand:** Validation, Writing – review & editing.

## Funding

Not declared.

Patient consent.

Patient accepted to participate at this study and signed consent for this study.

## Declaration of competing interest

The authors declare that they have no known competing financial interests or personal relationships that could have appeared to influence the work reported in this paper.

## References

[1] Fram B., Elder A., Namdari S. (2019). Periprosthetic humeral fractures in shoulder arthroplasty. JBJS Reviews.

[2] Sanchez-Sotelo J., Athwal G.S. (2022). Periprosthetic postoperative humeral fractures after shoulder arthroplasty. JAAOS.

[3] Canton G., Fazzari F., Fattori R., Ratti C., Murena L. (2019). Post-operative periprosthetic humeral fractures after reverse shoulder arthroplasty: a review of the literature. Acta Biomed.

[4] Mourkus H., Phillips N.J., Rangan A., Peach C.A. (2022). Management of periprosthetic fractures of the humerus: a systematic review. The Bone & Joint Journal.

[5] Trompeter A.J., Gupta R.R. (2013). The management of complex periprosthetic humeral fractures: a case series of strut allograft augmentation, and a review of the literature. Strategies Trauma Limb Reconstr.

[6] Rockwood C.A., Green D.P., Bucholz R.W., Heckman J.D., Court- Brown C.M. (2006).

[7] Steinmann S.P., Cheung E.V. (2008). Treatment of periprosthetic humerus fractures associated with shoulder arthroplasty. JAAOS.

[8] Bohsali K.I. (2006). Complications of total shoulder arthroplasty. J. Bone Joint Surg. Am..

[9] Cameron B., Iannotti J.P. (1999). Periprosthetic fractures of the humerus and scapula: management and prevention. Orthop. Clin. N. Am..

[10] Greiner S., Stein V., Scheibel M. (2011). Periprosthetic humeral fractures after shoulder and elbow arthroplasty. ACHOT.

[11] Kumar S., Sperling J.W., Haidukewych G.H., Cofield R.H. (2004). Periprosthetic humeral fractures after shoulder arthroplasty. JBJS.

[12] Martinez A.A., Calvo A., Cuenca J., Herrera A. (2011). Internal fixation and strut allograft augmentation for periprosthetic humeral fractures. J. Orthop. Surg. (Hong Kong).

[13] Rollo G., Biserni M., Huri G., Carulli C., Ronga M., Bisaccia M., Gomez-Garrido D., Ziroglu N., Bonura E.M., Ruberti A.A., Schiavone A., Meccariello L. (2020). Strut graft vs. traditional plating in the management of periprosthetic humeral fractures: a multicentric cohort study. Med. Glas..

[14] Thés A., Klouche S., de Tienda M., Bauer T., Hardy P. (2017). Cortical onlay strut allograft with cerclage wiring of periprosthetic fractures of the humerus without stem loosening: technique and preliminary results. Eur. J. Orthop. Surg. Traumatol..

[15] Vicenti G., Solarino G., Carrozzo M., Simone F., Ottaviani G., Bizzoca D., Zavattini G., Zaccari D., Buono C., Moretti B. (2022). Is the posterior approach with posterior locking compression plate and anterior allograft useful and safe in the treatment of periprosthetic humeral fractures following reverse total shoulder arthroplasty?. Geriatr. Orthop. Surg. Rehabil..

[16] Bonalive putty, Bonalive (n.d.). https://www.bonalive.com/en/products/bonalive-putty/ (accessed September 9, 2023).

